# Supplementary material for: Burkholderia pseudomallei BicA protein promotes pathogenicity in macrophages by regulating invasion, intracellular survival, and virulence
Source: mSphere. 2023 Sep 28;8(5):e00378-23. doi: 10.1128/msphere.00378-23 (PMC10597401; doi:10.1128/msphere.00378-23)

Figure S1: *ΔbicA* gene expression individual experiments. Gene expression from **Fig. 3** divided into individual experiments. Experiments 1 (A) and experiment 2 (B).

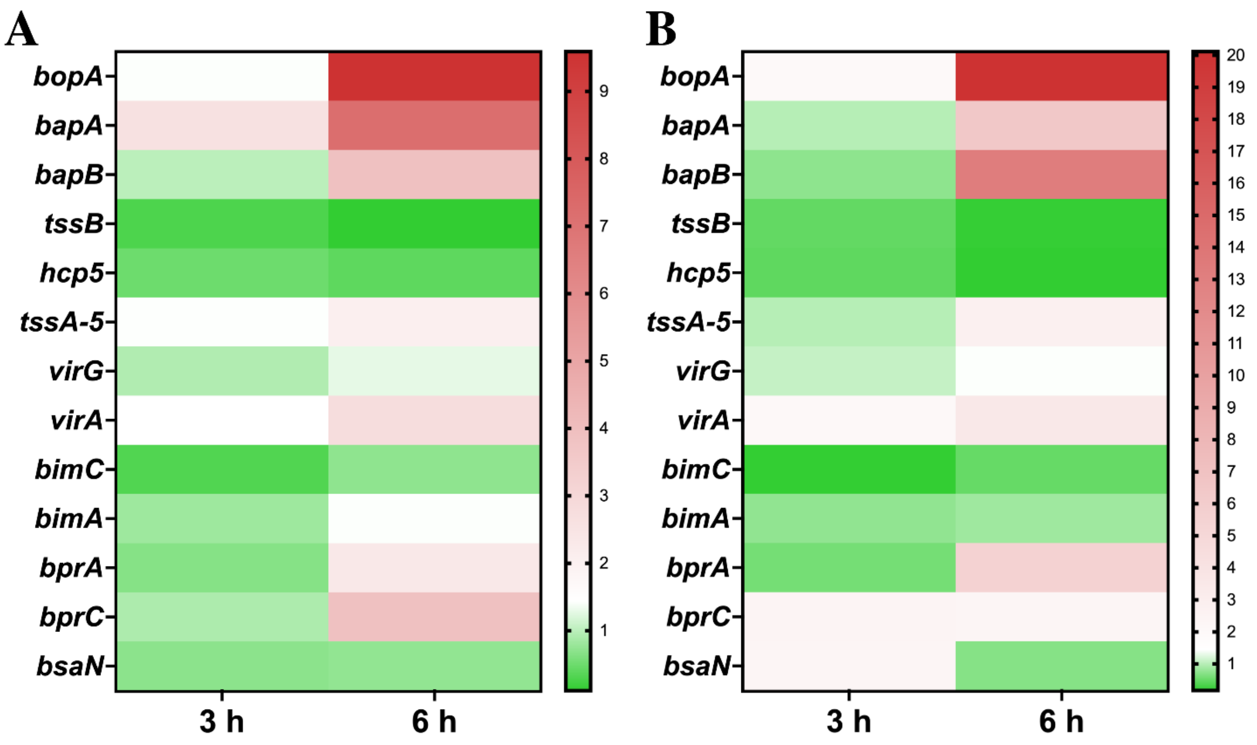

Supplement: Fig. S1 — ΔbicA gene expression individual experiments. [file msphere.00378-23-s0001.pdf]
