# Supplementary material for: Burkholderia pseudomallei BicA protein promotes pathogenicity in macrophages by regulating invasion, intracellular survival, and virulence
Source: mSphere. 2023 Sep 28;8(5):e00378-23. doi: 10.1128/msphere.00378-23 (PMC10597401; doi:10.1128/msphere.00378-23)

Figure S2: Flow cytometry gating strategy.

The gating strategy from [23] used to identify pulmonary macrophages.

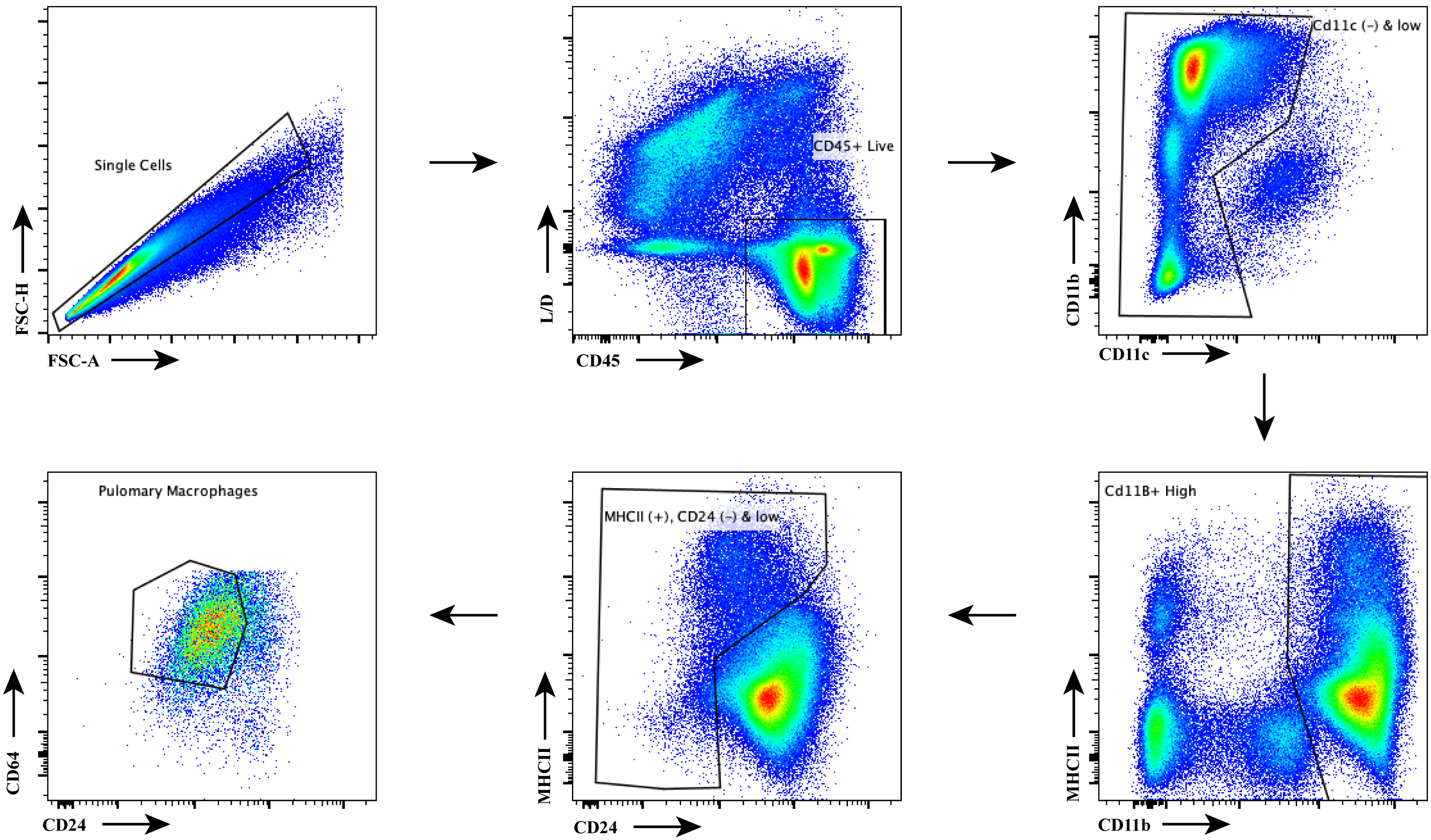

Supplement: Fig. S2 — Flow cytometry gating strategy. [file msphere.00378-23-s0002.pdf]
