# Supplementary material for: Burkholderia pseudomallei BicA protein promotes pathogenicity in macrophages by regulating invasion, intracellular survival, and virulence
Source: mSphere. 2023 Sep 28;8(5):e00378-23. doi: 10.1128/msphere.00378-23 (PMC10597401; doi:10.1128/msphere.00378-23)
Supplement: Table S1 — Fold change gene expression compared to WT at 3 and 6 hpi in RAW 264.7 cells. [file msphere.00378-23-s0003.pdf]

Table S1. Fold change gene expression compared to WT at 3 and 6 hpi in RAW 264.7 cells.

			Experiment 1					Experiment 2				
**Time point**	**Gene**	**Strain**	**Cq**	**Cq Mean**	**ΔCt 16S**	**ΔΔCt**	**$2^{-\Delta\Delta}$Ct**	**Cq**	**Cq Mean**	**ΔCt 16S**	**ΔΔCt**	**$2^{-\Delta\Delta}$Ct**
**3 h**	*bopA*	WT	27.16	31.78	13.61			31.04	31.48	14.13		
			32.14					32.01				
			31.42					31.39				
		Δ*bicA*	31.79	31.74	13.11	-0.503	**1.417**	31.48	31.05	13.17	-0.954	**1.938**
			32.26					30.59				
			31.16					31.08				
		Δ*bicA::bicA*	32.24	32.41	14.33	0.724	**0.605**	31.38	0.72	0.72	0.085	**0.943**
			32.28					31.93				
			32.69					30.34				
	bapA	WT	34.54	35.66	17.18			34.29	34.24	15.83		
			34.93					34.53				
			37.51					33.91				
		Δ*bicA*	33.88	34.78	15.80	-1.374	**2.592**	34.81	34.89	15.85	0.024	**0.984**
			34.49					35.24				
			35.97					34.62				
		Δ*bicA::bicA*	34.90	35.36	17.43	2.592	**0.841**	36.28	36.40	18.31	2.484	**0.179**
			35.59					35.56				
			35.58					37.36				
	bapB	WT	38.39	38.11	19.94			36.99	37.26	19.91		
			38.33					37.11				
			37.61					37.68				
		Δ*bicA*	38.29	38.58	19.95	0.008	**0.994**	38.48	38.23	20.36	0.446	**0.734**
			38.79					38.13				
			38.67					38.09				
		Δ*bicA::bicA*	37.97	38.83	20.76	0.814	**0.569**	38.48	37.98	20.98	1.070	**0.476**
			39.32					37.33				
			39.20					38.13				
	tssb	WT	33.24	33.41	14.93			34.63	33.85	15.44		
			33.40					33.55				
			33.59					33.36				

		Δ*bicA*	35.39	35.73	16.76	1.826	**0.282**	35.42	35.57	16.54	1.101	**0.466**
			36.07					37.01				
								34.29				
		Δ*bicA::bicA*	33.66	33.40	15.47	0.538	**0.689**	33.24	33.11	15.02	-0.412	**1.330**
			33.36					32.47				
			33.17					33.63				
	hcp	WT	31.18	31.29	13.12			30.70	30.33	12.98		
			31.37					29.83				
			31.32					30.44				
		Δ*bicA*	32.52	32.79	14.16	1.036	**0.488**	32.20	32.04	14.16	1.182	**0.441**
			32.66					32.14				
			33.18					31.77				
		Δ*bicA::bicA*	30.99	31.16	13.08	-0.035	**1.024**	29.64	29.64	12.64	-0.338	**1.264**
			30.57					29.35				
			31.91					29.93				
	tssA-5	WT	33.48	34.08	15.60			33.32	33.31	14.89		
			33.75					33.16				
			34.99					33.43				
		Δ*bicA*	34.64	34.06	15.09	-0.509	**1.423**	33.63	33.95	14.92	0.025	**0.983**
			33.49					34.00				
			34.06					34.23				
		Δ*bicA::bicA*	34.67	34.76	16.83	1.236	**0.424**	33.76	33.90	15.82	0.921	**0.528**
			35.19					34.36				
			34.42					33.59				
	virG	WT	32.78	32.61	14.13			33.47	32.86	14.45		
			32.53					32.44				
			32.53					32.68				
		Δ*bicA*	33.05	33.22	14.24	0.110	**0.926**	32.90	33.38	14.35	-0.108	**1.077**
			32.89					34.96				
			33.39					32.29				
		Δ*bicA::bicA*	35.10	33.51	15.58	1.448	**0.367**	32.39	32.53	14.44	-0.013	**1.009**
			32.73					32.02				
			32.70					33.17				

	virA	WT	34.69	33.80	15.32			34.53	33.89	15.48		
			33.40					33.52				
			33.30					33.60				
		Δ*bicA*	33.32	33.78	14.80	-0.517	**1.431**	33.70	33.48	14.45	-1.026	**2.037**
			34.33					0.00				
			33.68					33.27				
		Δ*bicA::bicA*	35.39	34.22	16.29	0.975	**0.509**	35.05	34.42	16.33	0.853	**0.554**
			33.69					34.39				
			33.58					33.81				
	bimC	WT	32.35	33.01	14.84			31.93	31.86	14.51		
			34.34					31.59				
			32.32					32.06				
		Δ*bicA*	36.15	35.16	16.53	1.695	**0.309**	35.28	34.95	17.07	2.561	**0.169**
								34.38				
			34.17					34.62				
		Δ*bicA::bicA*	34.09	34.09	16.01	1.178	**0.442**	33.97	33.07	16.07	1.562	**0.339**
			34.59					33.09				
			33.59					32.16				
	bimA	WT	31.40	31.86	13.69			31.38	31.51	14.16		
			32.75					31.80				
			31.42					31.34				
		Δ*bicA*	33.06	32.64	14.00	0.317	**0.803**	31.85	32.45	14.57	0.416	**0.749**
			31.97					33.33				
			32.88					32.16				
		Δ*bicA::bicA*	33.58	32.99	14.92	1.231	**0.426**	32.34	31.59	14.59	0.434	**0.740**
			31.81					30.96				
			33.58					31.47				
	bprA	WT	37.07	36.71	18.54			36.33	37.29	19.94		
			36.18					37.58				
			36.90					37.97				
		Δ*bicA*	38.16	37.80	19.17	0.624	**0.649**	38.13	38.61	20.73	0.788	**0.579**
			38.42					39.09				
			36.82									

		Δ*bicA::bicA*	40.03	38.37	20.30	1.756	**0.296**	36.82	36.73	19.73	-0.213	**1.159**
			37.74					36.51				
			37.35					36.87				
	bprC	WT	33.32	33.38	14.89			34.64	34.44	16.03		
			32.42					34.64				
			34.39					34.03				
		Δ*bicA*	34.08	34.02	15.05	0.153	**0.900**	33.57	33.78	14.74	-1.285	**2.436**
			34.47					33.62				
			33.52					34.14				
		Δ*bicA::bicA*	33.21	33.73	15.80	0.901	**0.535**	35.03	34.80	16.71	0.685	**0.622**
			34.03					35.92				
			33.94					33.45				
	bsaN	WT	36.01	37.05	18.57			39.12	38.06	19.65		
			38.09					37.01				
		Δ*bicA*	36.79	38.08	19.10	0.536	**0.689**	36.51	37.46	18.43	-1.223	**2.335**
			37.44					38.03				
			40.01					37.85				
		Δ*bicA::bicA*	35.84	36.20	18.27	-0.297	**1.228**	37.07	36.78	18.70	-0.956	**1.940**
			36.82					36.10				
			35.94					37.18				
**6 h**	*bopA*	WT	31.20	30.79	15.20			31.14	31.05	15.54		
			31.04					30.88				
			30.12					31.13				
		Δ*bicA*	31.90	31.72	11.93	-3.261	**9.588**	30.94	31.01	11.21	-4.330	**20.108**
			31.95					31.08				
			31.31					31.02				
		Δ*bicA::bicA*	30.05	30.49	14.00	-1.192	**2.285**	29.68	30.39	13.92	-1.621	**3.077**
			30.68					30.44				
			30.75					31.04				
	bapA	WT	35.19	34.80	18.56			35.90	35.06	18.47		
			34.72					35.04				
			34.49					34.24				

		bicA mutant	35.79	36.22	15.72	-2.841	**7.167**	36.71	36.80	15.79	-2.673	**6.378**
			35.43					0.00				
			37.44					36.88				
		Δ*bicA::bicA*	38.25	35.79	18.71	0.149	**0.902**	34.10	35.25	17.25	-1.219	**2.328**
			34.85					35.03				
			34.28					36.64				
	bapB	WT	38.12	37.33	21.74			36.25	36.79	21.28		
			36.97					36.76				
			36.91					37.35				
		Δ*bicA*	40.08	39.56	19.78	-1.960	**3.890**	36.98	37.36	17.56	-3.722	**13.194**
			39.05					37.44				
								37.66				
		Δ*bicA::bicA*	38.34	37.75	21.26	-0.484	**1.398**	37.11	37.22	20.75	-0.528	**1.442**
			37.58					37.15				
			37.32					37.39				
	tssb	WT	30.57	30.49	14.25			30.66	30.76	14.17		
			30.54					31.09				
			30.36					30.54				
		Δ*bicA*	36.62	37.96	17.46	3.203	**0.109**	38.58	37.64	16.63	2.461	**0.182**
			40.51					37.45				
			36.74					36.88				
		Δ*bicA::bicA*	30.65	31.00	13.92	-0.334	**1.260**	31.42	31.69	13.68	-0.490	**1.404**
			31.01					31.78				
			31.35					31.86				
	hcp	WT	27.35	27.29	11.70			27.21	27.24	11.73		
			27.24					27.32				
			27.28					27.19				
		Δ*bicA*	33.23	32.84	13.05	1.358	**0.390**	33.91	34.12	14.31	2.584	**0.167**
								34.32				
			32.44									
		Δ*bicA::bicA*	27.79	27.89	11.40	-0.293	**1.225**	27.76	27.69	11.22	-0.510	**1.424**
			27.76					27.63				
			28.13					27.67				

	tssA-5	WT	32.87	32.65	16.42			33.10	32.56	16.22		
			32.43					32.15				
			32.66					32.42				
		Δ*bicA*	34.54	35.88	15.38	-1.033	**2.047**	35.66	35.41	14.77	-1.448	**2.729**
			36.01					34.82				
			37.11					35.75				
		Δ*bicA::bicA*	32.20	32.62	15.53	-0.885	**1.847**	32.77	32.85	15.75	-0.472	**1.387**
			32.41					32.59				
			33.24					33.19				
	virG	WT	31.58	31.25	15.01			31.47	31.15	14.81		
			31.01					30.78				
			31.15					31.20				
		Δ*bicA*	34.89	35.16	14.66	-0.350	**1.274**	35.25	34.95	14.31	-0.503	**1.418**
			35.43					34.61				
								34.99				
		Δ*bicA::bicA*	31.57	31.35	14.26	-0.750	**1.682**	31.40	31.72	14.62	-0.193	**1.143**
			31.25					31.93				
			31.22					31.84				
	virA	WT	32.04	32.26	16.02			33.44	33.09	16.75		
			32.68					33.01				
			32.05					32.82				
		Δ*bicA*	35.86	35.04	14.54	-1.479	**2.787**	35.89	35.59	14.95	-1.799	**3.479**
			34.69					36.47				
			34.57					34.42				
		Δ*bicA::bicA*	33.05	32.97	15.88	-0.134	**1.097**	33.14	32.96	15.85	-0.899	**1.865**
			32.40					32.65				
			33.46					33.09				
	bimC	WT	30.57	30.50	14.91			30.73	30.73	15.22		
			30.92					30.96				
			30.02					30.50				
		Δ*bicA*	36.01	35.19	15.40	0.496	**0.709**	36.29	36.07	16.27	1.048	**0.484**
			34.37					36.07				
								35.86				

		Δ*bicA::bicA*	30.21	30.57	14.08	-0.830	**1.778**	31.03	31.22	14.75	-0.472	**1.387**
			30.75					30.88				
			30.74					31.74				
	bimA	WT	30.02	29.87	14.32			30.14	30.14	14.63		
			30.03					30.07				
			29.70					30.20				
		Δ*bicA*	34.06	33.83	13.82	-0.500	**1.415**	34.59	34.68	14.87	0.247	**0.842**
			33.45					33.91				
			33.30					35.54				
		Δ*bicA::bicA*	29.60	30.23	13.71	-0.609	**1.525**	29.89	30.12	13.65	-0.979	**1.972**
			30.25					30.06				
			30.75					30.40				
	bprA	WT	35.15	35.49	19.90			36.22	35.97	20.46		
			36.58					36.09				
			34.76					35.59				
		Δ*bicA*	38.35	38.46	18.68	-1.225	**2.337**	37.70	37.81	18.01	-2.450	**5.463**
			37.59					37.93				
			39.43									
		Δ*bicA::bicA*	35.42	36.13	19.64	-0.259	**1.197**	36.53	35.95	19.48	-0.975	**1.965**
			36.18					34.78				
			36.80					36.54				
	bprC	WT	33.02	32.37	16.14			32.19	32.16	15.82		
			32.15					32.35				
			31.95					31.94				
		Δ*bicA*	34.19	34.68	14.18	-1.960	**3.892**	34.30	35.25	14.61	-1.207	**2.308**
			34.35					35.58				
			35.50					35.88				
		Δ*bicA::bicA*	32.77	32.99	15.91	-0.231	**1.174**	32.21	32.39	15.28	-0.535	**1.449**
			33.81					32.44				
			32.39					32.51				
	bsaN	WT	35.60	37.17	20.93			37.60	38.04	21.70		
			36.21					38.48				
			39.69									

		Δ*bicA*	36.71	37.62	17.12	0.455	**0.730**	37.19	38.59	17.95	0.552	**0.682**
			37.23					38.33				
			38.93					40.25				
		Δ*bicA::bicA*	37.11	38.69	21.61	1.525	**0.348**	38.08	38.22	21.11	0.176	**0.885**
			38.12					33.63				
			40.85					38.35				
